# ﻿Identification and pathogenicity of *Aurifilum* species (Cryphonectriaceae, Diaporthales) on *Terminalia* species in Southern China

**DOI:** 10.3897/mycokeys.98.104719

**Published:** 2023-05-29

**Authors:** Wen Wang, ShuaiFei Chen

**Affiliations:** 1 Research Institute of Fast-Growing Trees (RIFT), Chinese Academy of Forestry (CAF), Zhanjiang 524022, China Chinese Academy of Forestry Zhanjiang China; 2 Ministry of Agricultural and Rural Affairs Key Laboratory of Molecular Biology of Crop Pathogens and Insect Pests, Institute of Biotechnology, Zhejiang University, Hangzhou 310058, China Zhejiang University Hangzhou China

**Keywords:** Cryphonectriaceae, fungal pathogen, Myrtle, pathogenicity, phylogenetic analysis

## Abstract

The family of Cryphonectriaceae (Diaporthales) contains many important tree pathogens and the hosts are wide-ranging. Tree species of *Terminalia* were widely planted as ornamental trees alongside city roads and villages in southern China. Recently, stem canker and cracked bark were observed on 2–6 year old *Terminalianeotaliala* and *T.mantaly* in several nurseries in Zhanjiang City, Guangdong Province, China. Typical conidiomata of Cryphonectriaceae fungi were observed on the surface of the diseased tissue. In this study, we used DNA sequence data (ITS, *BT2/BT1*, *TEF-1α*, *rpb2*) and morphological characteristics to identify the strains from Terminalia trees. Our results showed that isolates obtained in this study represent two species of *Aurifilum*, one previously described species, *A.terminali*, and an unknown species, which we described as *A.cerciana* sp. nov. Pathogenicity tests demonstrated that both *A.terminali* and *A.cerciana* were able to infect *T.neotaliala* and two tested *Eucalyptus* clones, suggesting the potential for *Aurifilum* fungi to become new pathogens of *Eucalyptus*.

## ﻿Introduction

Cryphonectriaceae is a fungal family within the order Diaporthales. This family is well-known for containing several species that are serious pathogens of trees, causing a wide range of diseases such as blight, die-back, and cankers (Gryzenhout et al. 2004, 2005, 2009; Begoude et al. 2010; Chen et al. 2010, 2011, 2013a, b, 2016, 2018; Wang et al. 2018, 2020; Roux et al. 2020). Most members of this family are easily recognizable based on the disease symptoms, as well as their distinctive yellow to orange or brown stromata and which can turn purple in 3% potassium hydroxide (KOH) and yellow in lactic acid (Gryzenhout et al. 2006, 2009; Jiang et al. 2020).

Twenty-four genera have been described in the Cryphonectriaceae (Gryzenhout et al. 2009, 2010; Begoude et al. 2010; Vermeulen et al. 2011, 2013; Crous et al. 2012; Chen et al. 2013a, b, 2016, 2018; Crane and Burgess 2013; Beier et al. 2015; Ali et al. 2018; Jiang et al. 2018, 2019, 2020; Ferreira et al. 2019; Wang et al. 2020; Huang et al. 2022). Some of the more well-known genera in this family include *Cryphonectriaparasitica*, which caused chestnut blight, and is one of the best-known tree-killing pathogen (Fairchild 1913; Shear and Stevens 1913; Anagnostakis 1987; Heiniger and Rigling 1994; Gryzenhout et al. 2009); *Chrysoportheaustroafricana* causes a canker disease of *Eucalyptus*, *Syzygium* and *Tibouchina* species in Southern and Eastern Africa (Wingfield et al. 1989; Gryzenhout et al. 2004; Roux et al. 2005; Nakabonge et al. 2006; Gryzenhout et al. 2009); *Chrysoporthecubensis* causes a canker disease of *Eucalyptus* species in West Africa and South America, and also causes diseases in Melastomataceae and Myrtaceae trees (Alfenas et al. 1983; Gryzenhout et al. 2004, 2009; Roux 2010); *Chrysoporthedeuterocubensis*, causes a canker disease of *Eucalyptus* species in Africa, Australia, China and Hawaii, and is also reported on native or non-native Melastomataceae and Myrtaceae trees (Davison and Coates 1991; Roux et al. 2005; Nakabonge et al. 2006; Zhou et al. 2008; Gryzenhout et al. 2009; Chen et al. 2010; Van der Merwe et al. 2010; Wang et al. 2020).

In China, various species of Cryphonectriaceae have been found to cause diseases in plants belonging to the Myrtales order. Some of the affected hosts include *Eucalyptus* hybrid (Chen et al. 2010, 2011; Wang et al. 2018, 2020), *Lagerstroemiaspeciosa* (*Lythraceae*, *Myrtales*) (Chen et al. 2018), *Melastomacandidum*, *M.sanguineum* (*Melastomataceae*, *Myrtales*), *Psidiumguajava* (Myrtaceae) (Chen et al. 2016; Wang et 2018, 2020), *Syzygiumcumini*, *S.hancei*, *S.jambos*, *S.samarangense* (Myrtaceae, Myrtales) (Chen et al. 2010, 2011; Van der Merwe et al. 2010; Wang et al. 2018, 2020), *Terminalianeotaliala* (Combretaceae) (Wang et al. 2020), *Rhodomyrtustomentosa* (Myrtaceae, Myrtales) (Chen et al. 2016). Inoculation tests have confirmed that all the Cryphonectriaceae species from Combretaceae, Lythraceae, Melastomataceae, and Myrtaceae in China are pathogenic to their original hosts and *Eucalyptus* (Chen et al. 2010, 2011, 2016, 2018; Wang et al. 2018, 2020).

Seven of the nine families of Myrtales are commonly found in southern China, and Cryphonectriaceae has been identified as an important pathogen to Myrtales trees in previous studies (Chen et al. 2010, 2011, 2016, 2018; Wang et al. 2018, 2020). Given the diverse climate and host range in southern China, there is potential for the discovery of various Cryphonectriaceae species and potential pathogens on Myrtales trees.

Terminalia species are economically and ecologically important trees in southern China and are widely used for timber, medicine, and ornamental purposes (Editorial Committee of Flora of China 1988; Batawila et al. 2005; Kamtchouing et al. 2006; Angiosperm Phylogeny Group 2009). In 2019, cankers were observed on the stems of Terminalia trees during disease surveys on Myrtales trees in southern China, and fruiting structures of the fungi on the cankered stems exhibited typical Cryphonectriaceae morphological characteristics. The aims of this study were to identify the fungi isolated from these cankers based on DNA sequencing and morphological characteristics and to test their pathogenicity on Terminalia species and two widely planted *E.grandis* hybrid genotypes.

## ﻿Materials and methods

### ﻿Disease symptoms, samples and isolations

In May 2019, disease surveys on Terminalia trees were conducted in Zhanjiang City, Guangdong Province in southern China. Sporocarps with typical characteristics of Cryphonectriaceae were observed on the surfaces of cankers on the branches, stems, and roots of Terminalia trees. In order to identify the pathogens, five experimental sites were set every 30 to 50 kilometers. Diseased bark pieces, branches, twigs, and roots bearing fruiting structures were collected and transported to the laboratory. The fruiting structures were incised using a sterile scalpel blade under a stereoscopic microscope. The spore masses were then transferred to 2% (v/v) malt extract agar (MEA) and incubated at room temperature for three to five days until colonies developed. The pure cultures were obtained by transferring single hyphal tips from the colonies to 2% MEA plates and incubated at room temperature for 7–10 days. The pure cultures are stored in the culture collection (CSF) at the Research Institute of Fast-Growing Trees (RIFT) (previous institution: China Eucalypt Research Centre, CERC), Chinese Academy of Forestry (CAF) in Zhanjiang, Guangdong Province, China.

### ﻿DNA extraction, polymerase chain reaction (PCR) amplification and sequencing

Representative isolates were selected for DNA sequence analyses, and actively growing mycelium on MEA cultures grown for one week at room temperature was scraped using a sterilized scalpel and transferred into 2.0 mL Eppendorf tubes. Total genomic DNA was extracted using the cetyltrimethylammonium bromide (CTAB) method described by Van Burik et al. (1998). The extracted DNA was dissolved in 30 μL TE buffer, and the concentration was measured using a Nano-Drop 2000 spectrometer (Thermo Fisher Scientific, Waltham, Massachusetts).

Based on previous research four gene regions, including internal transcribed spacer regions (ITS), two segments of β-tubulin (*BT2*/*BT1*), a partial segment of the translation elongation factor 1-α (*TEF-1α*) and RNA polymerase II (*rpb2*), were amplified and sequenced as described by Chen et al. (2010, 2016), Liu et al. (1999) and Jiang et al. (2022).

All amplified products were sequenced in both directions using the same primers that were used for the PCR amplification. Sequence reactions were performed by the Beijing Genomics Institute of Guangzhou, China. The nucleotide sequences were edited using Geneious 7.1.8 software. The sequences obtained in this study were submitted to GenBank (http://www.ncbi.nlm.nih.gov).

### ﻿Phylogenetic analysis

The preliminary identities of the isolates sequenced in this study were obtained by conducting a standard nucleotide BLAST search using the ITS, *BT2*, and *BT1* sequences. The BLAST results showed that the isolates collected in this study were mainly grouped in the genus *Aurifilum*. Phylogenetic analyses for strains identification in the current study were conducted for both genetic and species identification.

To determine the placement of *Aurifilum* species, two represent strains in this study were first determined by conducting phylogenetic analyses within Cryphonectriaceae species (Table 1) on combined datasets for the ITS and *BT2/BT1* regions. Then, the strains in the *Aurifilum* genus were further analyzed and identified using separate and combined datasets for the ITS, *BT2/BT1*, *TEF-1α*, and *rpb2* regions. Sequences of the *Aurifilum* isolates collected in this study and those from NCBI were aligned using MAFFT 7 (http://mafft.cbrc.jp/alignment/server) with the interactive refinement method (FFT-NS-i) setting (Katoh and Standley 2013). Then they were manually edited in MEGA X.

**Table 1.** Isolates from previous studies used in the phylogenetic analyses in the current study.

Identity	Isolate No.^a,b^	Host	Location	GenBank accession no.				
				*ITS*	*BT2*	*BT1*	*TEF*	*rpb2*
*Amphilogiagyrosa*	CMW10469T	*Elaeocarpusdentatus*	New Zealand	AF452111	AF525714	AF525707	MN271818	MN271782
	CMW10470	*Ela.dentatus*	New Zealand	AF452112	AF525715	AF525708	MN271819	MN271783
*Aurantioporthecorni*	MES1001	N/A	USA	KF495039	N/A	KF495069	N/A	N/A
	CTS1001	N/A	USA	KF495033	N/A	KF495063	N/A	N/A
	CMW10526	N/A	USA	DQ120762	AH015163	AH015163	N/A	N/A
*Aurantiosacculusacutatus*	CBS 132181T	*Eucalyptusviminalis*	Australia	JQ685514	N/A	N/A	MN271823	NA
*Aurantiosacculuscastaneae*	CFCC 52456	Castanea mollissima	China	MH514025	MH539688	MH539678	NA	MN271786
*Aurantiosacculuseucalyptorum*	CBS 130826T	*Euc.globulus*	Australia	JQ685515	N/A	N/A	MN271824	MN271785
*Aurapexpenicillata*	CMW10030T	*Miconiatheaezans*	Colombia	AY214311	AY214275	AY214239	N/A	N/A
	CMW10035	*Mic.theaezans*	Colombia	AY214313	AY214277	AY214241	N/A	N/A
*Aurifilummarmelostoma*	CBS124928T	*Terminaliamantaly*	Cameroon	FJ882855	FJ900590	FJ900585	MN271827	MN271788
	CBS124929	*Ter.ivorensis*	Cameroon	FJ882856	FJ900591	FJ900586	MN271828	MN271789
*Aurifilumterminali*	CSF10748	*Ter.neotaliala*	China	MN199834	MN258767	MN258772	MN258777	OQ942878
	CSF10757T	*Ter.neotaliala*	China	MN199837	MN258770	MN258775	MN258780	OQ942879
*Capillaureumcaryovora*	CBL02T	*Caryocarbrasiliense*	Brazil	MG192094	MG211808	MG211827	N/A	N/A
	CBL06	*Car.brasiliense*	Brazil	MG192096	MG211810	MG211829	N/A	N/A
*Celoportheborbonica*	CMW44128T	*Tibouchinagrandiflora*	La Réunion	MG585741	N/A	MG585725	N/A	N/A
	CMW44139	*Tib.grandiflora*	La Réunion	MG585742	N/A	MG585726	N/A	N/A
*Celoporthecerciana*	CERC 9128T	*Eucalyptus* hybrid tree 4	China, GuangDong	MH084352	MH084412	MH084382	MH084442	N/A
	CERC 9125	*Eucalyptus* hybrid tree 1	China, GuangDong	MH084349	MH084409	MH084379	MH084439	N/A
*Celoporthedispersa*	CMW 9976T	*Syzygiumcordatum*	South Africa	DQ267130	DQ267142	DQ267136	HQ730840	N/A
	CMW 9978	*S.cordatum*	South Africa	AY214316	DQ267141	DQ267135	HQ730841	N/A
*Celoportheeucalypti*	CMW 26900	*Eucalyptus* clone EC48	China	HQ730836	HQ730826	HQ730816	HQ730849	N/A
	CMW 26908T	*Eucalyptus* clone EC48	China	HQ730837	HQ730827	HQ730817	HQ730850	N/A
*Celoporthefontana*	CMW 29375	*S.guineense*	Zambia	GU726940	GU726952	GU726952	JQ824073	N/A
	CMW 29376T	*S.guineense*	Zambia	GU726941	GU726953	GU726953	JQ824074	N/A
*Celoportheguangdongensis*	CMW 12750T	*Eucalyptus* sp.	China	HQ730830	HQ730820	HQ730810	HQ730843	N/A
*Celoportheindonesiensis*	CMW 10781T	*S.aromaticum*	Indonesia	AY084009	AY084021	AY084033	HQ730842	N/A
*Celoporthesyzygii*	CMW 34023T	*S.cumini*	China	HQ730831	HQ730821	HQ730811	HQ730844	N/A
	CMW24912	*S.cumini*	China	HQ730833	HQ730823	HQ730813	HQ730846	N/A
*Celoporthetibouchineae*	CMW44126T	*Tib.grandiflora*	La Réunion	MG585747	N/A	MG585731	N/A	N/A
	CMW44127	*Tib.grandiflora*	La Réunion	MG585748	N/A	MG585732	N/A	N/A
*Celoporthewoodiana*	CMW13936T	*Tib.granulosa*	South Africa	DQ267131	DQ267143	DQ267137	JQ824071	N/A
	CMW13937	*Tib.granulosa*	South Africa	DQ267132	DQ267144	DQ267138	JQ824072	N/A
*Chrysomorbuslagerstroemiae*	CERC 8780	*Lagerstroemiaspeciosa*	China	KY929330	KY929340	KY929350	N/A	N/A
	CERC 8810T	*Lag.speciosa*	China	KY929338	KY929348	KY929358	N/A	N/A
*Chrysoportheaustroafricana*	CMW 62	*Euc.grandis*	South Africa	AF292041	AF273458	AF273063	N/A	N/A
	CMW 9327	*Tib.granulosa*	South Africa	AF273473	AF273455	AF273060	N/A	N/A
	CMW 2113T	*Euc.grandis*	South Africa	AF046892	AF273462	AF273067	N/A	N/A
*Chrysoporthecubensis*	CMW 10453	*Euc.saligna*	Democratic Republic of the Congo	AY063476	AY063480	AY063478	N/A	N/A
	CMW 10669 = CRY864	*Eucalyptus* sp.	Republic of the Congo	AF535122	AF535126	AF535124	N/A	N/A
*Chrysoporthedeuterocubensis*	CMW 11290	*Eucalyptus* sp.	Indonesia	AY214304	AY214268	AY214232	N/A	N/A
	CMW 8651	*S.aromaticum*	Indonesia	AY084002	AY084014	AY084026	N/A	N/A
*Chrysoporthedoradensis*	CMW 11287T	*Euc.grandis*	Ecuador	AY214289	AY214253	AY214217	N/A	N/A
	CMW 11286	*Euc.grandis*	Ecuador	AY214290	AY214254	AY214218	N/A	N/A
*Chrysoporthehodgesiana*	CMW 10625	*Mic.theaezans*	Colombia	AY956970	AY956980	AY956979	N/A	N/A
	CMW 9995	*Tib.semidecandra*	Colombia	AY956969	AY956978	AY956977	N/A	N/A
	CMW 10641T	*Tib.semidecandra*	Colombia	AY692322	AY692325	AY692326	N/A	N/A
*Chrysoportheinopina*	CMW 12727T	*Tib.lepidota*	Colombia	DQ368777	DQ368807	DQ368806	N/A	N/A
	CMW 12729	*Tib.lepidota*	Colombia	DQ368778	DQ368809	DQ368808	N/A	N/A
*Chrysoporthesyzygiicola*	CMW 29940T	*S.guineense*	Zambia	FJ655005	FJ805236	FJ805230	N/A	N/A
	CMW 29942	*S.guineense*	Zambia	FJ655007	FJ805238	FJ805232	N/A	N/A
*Chrysoporthezambiensis*	CMW29928T	*Euc.grandis*	Zambia	FJ655002	FJ805233	FJ858709	N/A	N/A
	CMW29930	*Euc.grandis*	Zambia	FJ655004	FJ805235	FJ858711	N/A	N/A
*Corticimorbussinomyrti*	CERC3629T	*Rhodomyrtustomentosa*	China	KT167169	KT167189	KT167189	N/A	N/A
	CERC3631	*Rho.tomentosa*	China	KT167170	KT167190	KT167190	N/A	N/A
*Cryphonectriacitrina*	CBS 109758	*Punicagranatum*	USA	MN172407	N/A	N/A	MN271843	EU219342
*Cryphonectriadecipiens*	CMW 10436	*Quercussuber*	Portugal	AF452117	AF525710	AF525703	N/A	N/A
	CMW 10484	*Castaneasativa*	Italy	AF368327	AF368349	AF368349	N/A	N/A
*Cryphonectriajaponica*	CMW13742	*Q.grosseserrata*	Japan	AY697936	AY697962	AY697961	N/A	N/A
*Cryphonectriamacrospora*	CMW10463	*Cas.cuspidata*	Japan	AF368331	AF368350	AF368351	N/A	N/A
	CMW10914	*Cas.cuspidata*	Japan	AY697942	AY697974	AY697973	N/A	N/A
*Cryphonectrianaterciae*	C0612	*Q.suber*	Portugal	EU442657	N/A	N/A	MN271844	MN271796
*Cryphonectrianeoparasitica*	CFCC 52146	*Cas.mollissima*	China	MH514029	MH539692	MH539682	MH539693	N/A
*Cryphonectriaparasitica*	CMW 7048	*Q.virginiana*	USA	AF368330	AF273470	AF273076	MF442684	N/A
	CMW 13749	*Cas.mollisima*	Japan	AY697927	AY697944	AY697943	N/A	N/A
*Cryphonectriaquercicola*	CFCC 52140T	*Q.wutaishansea*	China, Shaanxi	MG866026	MG896113	MG896117	N/A	N/A
	CFCC 52141	*Q.wutaishansea*	China, Shaanxi	MG866027	MG896114	MG896118	N/A	N/A
*Cryphonectriaquercus*	CFCC 52138T	*Q. aliena var. acuteserrata*	China, Shaanxi	MG866024	MG896111	MG896115	MN271849	N/A
	CFCC 52139	*Q. aliena var. acuteserrata*	China, Shaanxi	MG866025	MG896112	MG896116	N/A	N/A
*Cryphonectriaradicalis*	CMW10455	*Q.suber*	Italy	AF452113	AF525712	AF525705	N/A	N/A
	CMW 10477	*Q.suber*	Italy	AF368328	AF368347	AF368347	N/A	N/A
	CMW 13754	*Fagusjaponica*	Japan	AY697932	AY697954	AY697953	N/A	N/A
*Cryptometrionaestuescens*	CMW18793	*Euc.grandis*	Indonesia	GQ369459	GQ369456	GQ369456	N/A	N/A
	CMW28535T	*Euc.grandis*	North Sumatra, Indonesia	GQ369457	GQ369454	GQ369454	N/A	N/A
*Diversimorbusmetrosiderotis*	CMW37321	*Metrosiderosangustifolia*	South Africa	JQ862870	JQ862952	JQ862911	N/A	N/A
	CMW37322T	*Met.angustifolia*	South Africa	JQ862871	JQ862953	JQ862912	N/A	N/A
*Endothiacerciana*	CSF 15398	*Quercus* sp.	China	OM801201	OM685050	OM685038	N/A	N/A
	CSF 15420	*Quercus* sp.	China	OM801208	OM685033	OM685045	N/A	N/A
*Endothiachinensis*	CFCC 52144	*C.mollissima*	China	MH514027	MH539690	MH539680	MN271860	N/A
	CMW2091	*Q.palustris*	USA	AF368325	AF368336	AF368337	N/A	N/A
	CMW10442	*Q.palustris*	USA	AF368326	AF368338	AF368339	N/A	N/A
*Holocryphiacapensis*	CMW37887T	*Met.angustifolia*	South Africa	JQ862854	JQ862936	JQ862895	JQ863051	N/A
	CMW37329	*Met.angustifolia*	South Africa	JQ862859	JQ862941	JQ862900	JQ863056	N/A
*Holocryphiaeucalypti*	CMW7033T	*Euc.grandis*	South Africa	JQ862837	JQ862919	JQ862878	JQ863034	N/A
	CMW7035	*Euc.saligna*	South Africa	JQ862838	JQ862920	JQ862879	JQ863035	N/A
*Holocryphiagleniana*	CMW37334T	*Met.angustifolia*	South Africa	JQ862834	JQ862916	JQ862875	JQ863031	N/A
	CMW37335	*Met.angustifolia*	South Africa	JQ862835	JQ862917	JQ862876	JQ863032	N/A
*Holocryphiamzansi*	CMW37337T	*Met.angustifolia*	South Africa	JQ862841	JQ862923	JQ862882	JQ863038	N/A
	CMW37338	*Met.angustifolia*	South Africa	JQ862842	JQ862924	JQ862883	JQ863039	N/A
*Holocryphia* sp.	CMW6246	*Tib.granulosa*	Australia	JQ862845	JQ862927	JQ862886	JQ863042	N/A
*Holocryphia* sp.	CMW10015	*Euc.fastigata*	New Zealand	JQ862849	JQ862931	JQ862890	JQ863046	N/A
*Immersiportheknoxdaviesiana*	CMW37314T	*Rapaneamelanophloeos*	South Africa	JQ862765	JQ862775	JQ862785	N/A	N/A
	CMW37315	*Rap.melanophloeos*	South Africa	JQ862766	JQ862776	JQ862786	N/A	N/A
*Latruncellusaurorae*	CMW28274	*Galpiniatransvaalica*	Swaziland	GU726946	GU726958	GU726958	N/A	N/A
	CMW28276T	*G.transvaalica*	Swaziland	GU726947	GU726959	GU726959	N/A	N/A
*Luteocirrhusshearii*	CBS130775	*Banksiabaxteri*	Australia	KC197024	KC197009	KC197015	N/A	N/A
	CBS130776T	*B.baxteri*	Australia	KC197021	KC197006	KC197012	N/A	N/A
*Microthiahavanensis*	CMW11301	*Myr.faya*	Azores	AY214323	AY214287	AY214251	N/A	N/A
	CMW14550	*E.saligna*	Mexico	DQ368735	DQ368742	DQ368741	N/A	N/A
*Myrtonectriamyrtacearum*	CMW46433T	*Heteropyxisnatalensis*	South Africa	MG585736	MG585734	MG585720	N/A	N/A
	CMW46435	*S.cordatum*	South Africa	MG585737	MG585735	MG585721	N/A	N/A
*Parvosmorbuseucalypti*	CERC2060	*Eucalyptus* hybrid clone	China	MN258787	MN258801	MN258815	MN258829	N/A
	CERC2061T	*Eucalyptus* hybrid clone	China	MN258788	MN258802	MN258816	MN258830	N/A
*Parvosmorbusguangdongensis*	CERC10459	*E.urophylla* hybrid clone	China	MN258798	MN258812	MN258826	MN258840	N/A
	CERC10460T	*E.urophylla* hybrid clone	China	MN258799	MN258813	MN258827	MN258841	N/A
*Pseudocryphonectriaelaeocarpicola*	CFCC 57515	*Elaeocarpus* spp.	China	ON489048	N/A	N/A	ON456916	ON456918
	CFCC 57516	*Elaeocarpus* spp.	China	ON489049	N/A	N/A	ON456917	ON456919
*Rostraureumtropicale*	CMW9972	*Ter.ivorensis*	Ecuador	AY167436	AY167431	AY167426	N/A	N/A
	CMW10796T	*Ter.ivorensis*	Ecuador	AY167438	AY167433	AY167428	N/A	N/A
*Ursicollumfallax*	CMW18119T	*Coccolobauvifera*	USA	DQ368755	DQ368759	DQ368758	N/A	N/A
	CMW18115	*Coc.uvifera*	USA	DQ368756	DQ368761	DQ368760	N/A	N/A
*Diaportheambigua*	CMW5587	*Malusdomestica*	South Africa	AF543818	AF543822	AF543820	N/A	N/A

^a^ Designation of isolates and culture collections: CMW = Tree Protection Co-operative Program, Forestry and Agricultural Biotechnology Institute, University of Pretoria, South Africa; ATCC = American Type Culture Collection, Manassas, USA; MES, CTS represent isolates in Beier et al. 2015; CBS = Westerdijk Fungal Biodiversity Institute, Utrecht, Netherlands; CBL represent isolates in Ferreira et al. 2019; CERC = China Eucalypt Research Centre (CERC), Chinese Academy of Forestry (CAF), ZhanJiang, GuangDong, China; CFCC = China Forestry Culture Collection Center, Beijing, China. ^b^ “T” following isolate number means isolates are ex-type or from samples that have been linked morphologically to type material of the species. ^c^ N/A = not available.

The taxonomic positions of two methods were used for phylogenetic analyses. Maximum parsimony (MP) analyses were performed using PAUP v. 4.0 b10 (Swofford 2003) and maximum likelihood (ML) analyses were conducted with PhyML v. 3.0 (Guindon and Gascuel 2003).

For MP analyses, gaps were treated as a fifth character, and characters were unordered and of equal weight with 1,000 random addition replicates. A partition homogeneity test (PHT) using PAUP v. 4.0 b10 (Swofford 2003) was conducted to determine whether data for the four genes could be combined. The most parsimonious trees were obtained using the heuristic search option with stepwise addition, tree bisection, and reconstruction branch swapping. MAXTREES was set to 5,000 and zero-length branches collapsed. A bootstrap analysis (50% majority rule, 1,000 replicates) was carried out to determine statistical support for internal nodes in trees. Tree length (TL), consistency index (CI), retention index (RI) and homoplasy index (HI) were used to assess phylogenetic trees (Hillis and Huelsenbeck 1992).

For ML analyses, the best nucleotide substitution model for each dataset was established using jModeltest v. 2.1.5 (Posada 2008). In PhyML, the maximum number of retained trees was set to 1,000 and nodal support was determined by non-parametric bootstrapping with 1,000 replicates. For both MP and ML analyses, the phylogenetic trees were viewed using MEGA v. 6.0.

### ﻿Morphology

The representative isolates identified as the new species by DNA sequence analysis were grown on 2% water ager (WA), to which sterilized freshly cut branch sections (0.5–1 cm diam. 4–5 cm length) of *Eucalyptusurophylla* × *E.grandis* (CEPT53) branch sections were added. These fungi with branch sections on 2% WA were incubated at room temperature for 6–8 wks until fruiting structures emerged. Representative cultures are maintained in the China General Microbiological Culture Collection Centre (CGMCC), Beijing, China. Isolates linked to the type specimens connected to representative isolates were deposited in the mycological fungarium of the Institute of Microbiology, Chinese Academy of Sciences (HMAS), Beijing, China, and the Collection of Central South Forestry Fungi of China (CSFF), Guangdong Province, China.

The structures that emerged on the surface of the *Eucalyptus* branches were mounted in one drop of 85% lactic acid on glass slides under a dissecting microscope and then embedded in Leica Bio-systems Tissue Freezing Medium (Leica Biosystems nussloch GmbH, Nussloch, Germany) and sectioned (6 μm thick) using a Microtome Cryostat Microm HM550 (Microm International GmbH, Thermo Fisher Scientific, Walldorf, Germany) at -20 °C. Conidiophores, conidiogenous cells, and conidia were measured after crushing the sporocarps on microscope slides in sterilized water. For the holotype specimens, 50 measurements were performed for each morphological feature, and 30 measurements per character were made for the remaining specimens.

Measurements were recorded using an Axio Imager A1 microscope (Carl Zeiss Ltd., Munchen, Germany) and an AxioCam ERc 5S digital camera with Zeiss Axio Vision Rel. 4.8 software (Carl Zeiss Ltd., Munchen, Germany). The results are presented as (minimum–) (mean – standard deviation) – (mean + standard deviation) (–maximum).

Isolates identified as new species were selected for studying culture characteristics. After the isolates were grown for 7 days on 2% MEA, a 5 mm plug was removed from each culture and transferred to the central of 90 mm MEA Petri dishes. The cultures were incubated in the dark under temperatures ranging from 5 °C to 35 °C at 5 °C intervals. Five replicate plates for each isolate at each temperature condition were prepared. Two diameter measurements, perpendicular to each other, were taken daily for each colony until the fastest-growing culture had covered the 90 mm Petri dishes. Averages of the diameter measurements at each of the seven temperatures were computed with Microsoft Excel 2016 (Microsoft Corporation, Albuquerque, NM, USA). Colony colors were determined by incubating the isolates on fresh 2% MEA at 25 °C in the dark after 7 days. The color descriptions of the sporocarps and colonies were according to the color charts of Rayner (1970).

### ﻿Pathogenicity tests

In this study, inoculations were conducted on two different *Eucalyptus* hybrid genotypes (CEPT46 and CEPT53) and *T.neotaliala* to understand the pathogenicity on *Eucalyptus* plantations and to fulfill Koch’s postulates. The selected isolates were grown on 2% MEA at 25 °C for 10 days before inoculation. Each selected isolate was inoculated on 10 seedlings or branches of each inoculated tree, and 10 additional seedlings or branches were inoculated with sterile MEA plugs to serve as negative controls. The inoculations were conducted in August 2019, and the results were evaluated after 7 weeks by measuring the lengths of the lesions on the cambium.

Inoculations were conducted on *T.mantaly* and two widely planted *E.grandis* hybrid genotype (CEPT46, CEPT53) to fulfill Koch’s postulates and understand the pathogenicity on *Eucalyptus* plantations. The selected isolates were grown on 2% MEA at 25 °C for 10 d before inoculation. Each of the selected isolates was inoculated on 10 seedlings or branches of each selected tree variety, and 10 additional seedlings or branches were inoculated with sterile MEA plugs to serve as negative controls. The inoculations on seedlings of two 1-year-old *Eucalyptus* hybrid genotypes were conducted in the glasshouse, and the inoculations on branches of 10-year-old *T.mantaly* were conducted in the field. The inoculations method followed Chen et al. (2010, 2013b).

Inoculations were conducted in August 2019 and the results were evaluated after 7 weeks by measuring the lengths (mm) of the lesions on the cambium. For re-isolations, small pieces of discolored xylem from the edges of the resultant lesions were cut and placed on 2% MEA at room temperature. Re-isolations of all seedlings/branches inoculated as negative controls and from four randomly selected trees per isolate were conducted. The identities of the re-isolated fungi were confirmed by morphological comparisons. The inoculation results were analyzed using SPSS Statistics 26 software (BM Corp., Armonk, NY, USA) by one-way analysis of variance (ANOVA).

## ﻿Results

### ﻿Isolation

**Figure 1.**
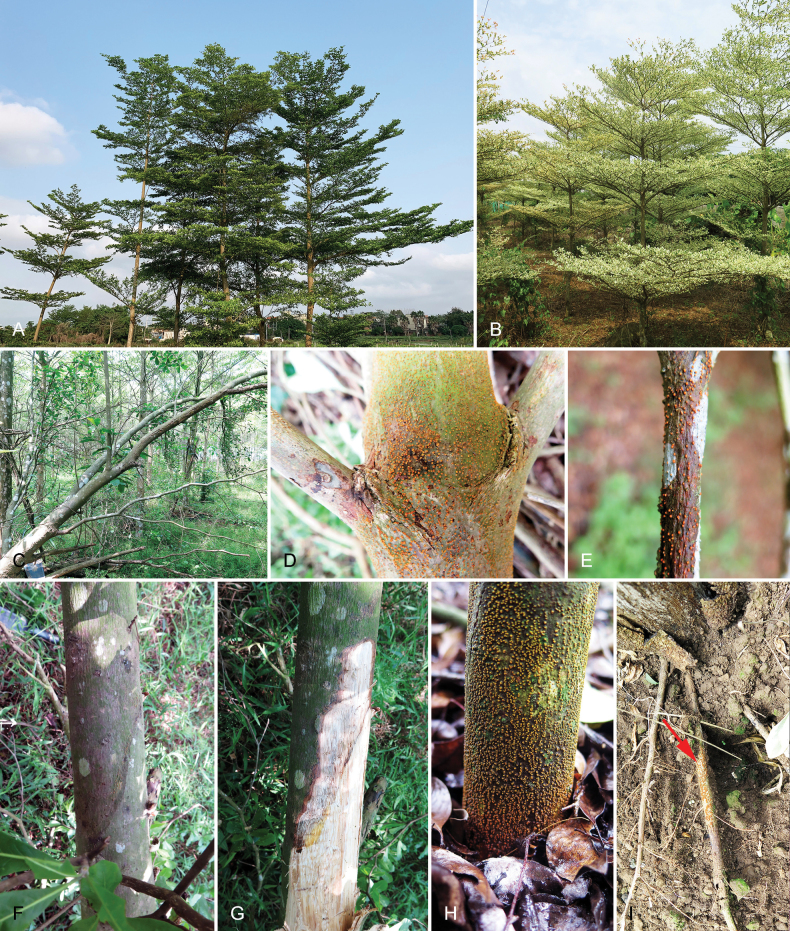
Disease symptoms on Terminalia trees associated with infection by *Aurifilum* spp. **A***Terminalianeotaliala* in the field **B***Terminaliamantaly* in a nursery **C** the main stems and branches of *T.neotaliala* infected by *Aurifilum* species and resulted in tree death **D, E** sporocarps of *Aurifilum* species on the main stem of *T.neotaliala* (**D**), and branch of *T.mantaly* (**E**) **F, G** lesions developing on the branches of *T.neotaliala***H, I** Sporocarps of *Aurifilum* species on the base of main stem (**H**) and roots of *T.neotaliala* (**I**).

Diseased samples from 14 trees were collected from three sites (20190523-1, 20190525-2, 20190525-3) of *T.neotaliala* (Fig. 1A) nurseries, and two sites (20190525-1, 20190525-4) of *T.mantaly* (Fig. 1B) nurseries (Table 2). In the surveyed sites, 10%–25% of Terminalia trees were infected. Cankers with stromata on the main stem bark surface, which often resulted in tree death, were observed on two to five-year-old *T.neotaliala* trees (Fig. 1C). Obvious orange conidiomata were observed on the branches and twigs of three-year-old *T.mantaly* trees (Fig. 1D, E). Developing lesions were observed on the main stem of *T.neotaliala* and resulting in bark depression (Fig. 1F) and xylem necrosis (Fig. 1G). Orange fruiting structures even presented on the barks of the main stem base (Fig. 1H) and roots (Fig. 1I). The fruiting structures on *T.neotaliala* and *T.mantaly* displayed the typical morphological characteristics of Cryphonectriaceae (Gryzenhout et al. 2009; Wang et al. 2020). Isolates obtained from the asexual fruiting structures on MEA were white when young and turned yellow with age, and the isolates on MEA exhibited typical morphological characteristics of Cryphonectriaceae. Twenty isolates from both *T.neotaliala* and *T.mantaly* in the five sampled nurseries were isolated and sequenced for further studies (Table 2).

**Table 2.** Isolates sequenced and used for phylogenetic analyses, morphological studies and pathogenicity tests in the current study.

Identity	Isolate Number	Genotype^a^	Host	Nursery No.	Location	GPS iformation	Collector	GenBank accession No.					References
								*ITS*	*tub2*	*tub1*	*tef1*	*rpb2*	
*A.terminali*	CSF16295	AAAAA	*T.neotaliala*	20190523-1	ChaTing, LingBei, SuiXi	21°16′06.97"N, 110°5′16.8432"E	S.F.Chen & W. Wang	OQ912905	OQ921705	OQ921623	OQ921643	OQ921663	This study
*A.terminali*	CSF16309	AAAAA	*T.mantaly*	20190525-1	DaJia, SuiCheng, SuiXi	21°18′44.19"N, 110°11′46.7268"E	S.F.Chen & W. Wang	OQ912906	OQ921706	OQ921624	OQ921644	OQ921664	This study
*A.terminali*	CSF16310^d^	AAAAA	*T.mantaly*	20190525-1	DaJia, SuiCheng, SuiXi	21°18′44.19"N, 110°11′46.7268"E	S.F.Chen & W. Wang	OQ912907	OQ921707	OQ921625	OQ921645	OQ921665	This study
*A.terminali*	CSF16356^d^	AAAAA	*T.neotaliala*	20190525-3	DiaoLou, LingBei, SuiXi	21°15′57.006"N, 110°12′26.5824"E	S.F.Chen & W. Wang	OQ912908	OQ921708	OQ921626	OQ921646	OQ921666	This study
*A.terminali*	CSF16377	AAAAA	*T.mantaly*	20190525-4	DiaoLou, LingBei, SuiXi	21°15′57.006"N, 110°12′26.5824"E	S.F.Chen & W. Wang	OQ912909	OQ921709	OQ921627	OQ921647	OQ921667	This study
*A.terminali*	CSF16380	AAAAA	*T.mantaly*	20190525-4	DiaoLou, LingBei, SuiXi	21°15′57.006"N, 110°12′26.5824"E	S.F.Chen & W. Wang	OQ912910	OQ921710	OQ921628	OQ921648	OQ921668	This study
*A.terminali*	CSF16343^d^	AABAA	*T.neotaliala*	20190525-2	DuHao, MaZhang, MaZhang	21°14′16.4076"N, 110°17′23.9964"E	S.F.Chen & W. Wang	OQ912911	OQ921711	OQ921629	OQ921649	OQ921669	This study
*A.terminali*	CSF16387	AABAA	*T.mantaly*	20190525-4	DiaoLou, LingBei, SuiXi	21°15′57.006"N, 110°12′26.5824"E	S.F.Chen & W. Wang	OQ912912	OQ921712	OQ921630	OQ921650	OQ921670	This study
*A.terminali*	CSF16388^d^	AABAA	*T.mantaly*	20190525-4	DiaoLou, LingBei, SuiXi	21°15′57.006"E 110°12′26.5824"E	S.F.Chen & W. Wang	OQ912913	OQ921713	OQ921631	OQ921651	OQ921671	This study
*A.cerciana*	**CSF16384^c, d^ = CGMCC3.20108**	BBCBB	*T.mantaly*	20190525-4	DiaoLou, LingBei, SuiXi	21°15′57.006"N, 110°12′26.5824"E	S.F.Chen & W. Wang	OQ912914	OQ921714	OQ921632	OQ921652	OQ921672	This study
*A.cerciana*	CSF16250	BBCBB	*T.neotaliala*	20190523-1	ChaTing, LingBei, SuiXi	21°16′06.97"N, 110°5′16.8432"E	S.F.Chen & W. Wang	OQ912915	OQ921715	OQ921633	OQ921653	OQ921673	This study
*A.cerciana*	CSF16251	BBCBB	*T.neotaliala*	20190523-1	ChaTing, LingBei, SuiXi	21°16′06.97"N, 110°5′16.8432"E	S.F.Chen & W. Wang	OQ912916	OQ921716	OQ921634	OQ921654	OQ921674	This study
*A.cerciana*	**CSF16261^b, c, d^ = CGMCC3.20107**	BBCBB	*T.neotaliala*	20190523-1	ChaTing, LingBei, SuiXi	21°16′06.97"N, 110°5′16.8432"E	S.F.Chen & W. Wang	OQ912917	OQ921717	OQ921635	OQ921655	OQ921675	This study
*A.cerciana*	CSF16262	BBCBB	*T.neotaliala*	20190523-1	ChaTing, LingBei, SuiXi	21°16′06.97"N, 110°5′16.8432"E	S.F.Chen & W. Wang	OQ912918	OQ921718	OQ921636	OQ921656	OQ921676	This study
*A.cerciana*	CSF16267	BBCBB	*T.neotaliala*	20190523-1	ChaTing, LingBei, SuiXi	21°16′06.97"N, 110°5′16.8432"E	S.F.Chen & W. Wang	OQ912919	OQ921719	OQ921637	OQ921657	OQ921677	This study
*A.cerciana*	CSF16268	BBCBB	*T.neotaliala*	20190523-1	ChaTing, LingBei, SuiXi	21°16′06.97"N, 110°5′16.8432"E	S.F.Chen & W. Wang	OQ912920	OQ921720	OQ921638	OQ921658	OQ921678	This study
*A.cerciana*	CSF16273	BBCBB	*T.neotaliala*	20190523-1	ChaTing, LingBei, SuiXi	21°16′06.97"N, 110°5′16.8432"E	S.F.Chen & W. Wang	OQ912921	OQ921721	OQ921639	OQ921659	OQ921679	This study
*A.cerciana*	CSF16385	BBCBB	*T.mantaly*	20190525-4	DiaoLou, LingBei, SuiXi	21°15′57.006"N, 110°12′26.5824"E	S.F.Chen & W. Wang	OQ912922	OQ921722	OQ921640	OQ921660	OQ921680	This study
*A.cerciana*	CSF16351^c, d^	BBCBC	*T.neotaliala*	20190525-3	DiaoLou, LingBei, SuiXi	21°15′57.006"N, 110°12′26.5824"E	S.F.Chen & W. Wang	OQ912923	OQ921723	OQ921641	OQ921661	OQ921681	This study
*A.cerciana*	CSF16352^c, d^	BBCBC	*T.neotaliala*	20190525-3	DiaoLou, LingBei, SuiXi	21°15′57.006"N, 110°12′26.5824"E	S.F.Chen & W. Wang	OQ912924	OQ921724	OQ921642	OQ921662	OQ921682	This study

^a^ Genotype determined by sequence of ITS, *tub2*, *tub1*, *tef1*, and *rpb2* four regions. ^b^ Isolates ex-type. ^c^ Isolates used for culture growth. ^d^ Isolates used in pathogenicity.

### ﻿Phylogenetic analysis

**Figure 2.**
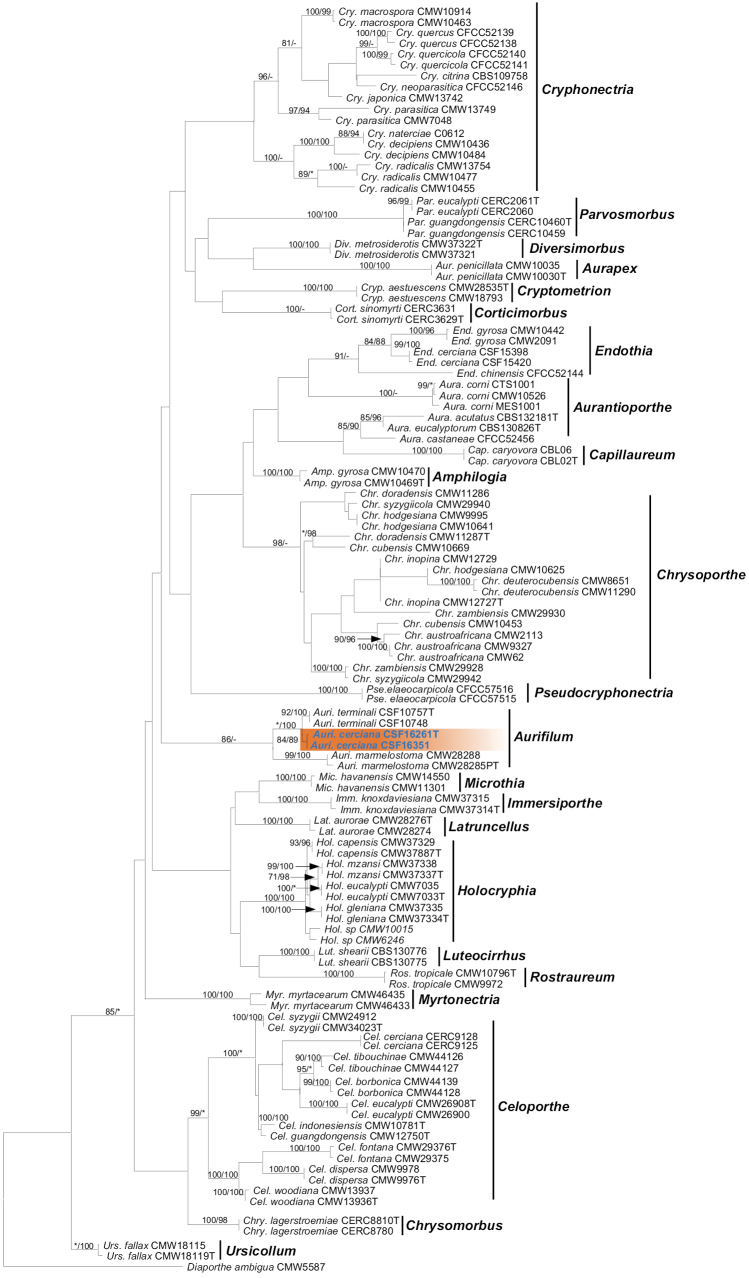
Phylogenetic trees based on maximum likelihood (ML) analyses of combined DNA sequence dataset of combination of ITS and BT2/BT1 regions for species in Cryphonectriaceae. combination of, *TEF-1α* and *rpb2* regions. Bootstrap values ≥ 70% for ML and MP (maximum parsimony) analyses are presented at branches as ML/MP. Bootstrap value lower than 70% are marked with *, and absent analysis value are marked with –. Isolates representing *Aurifilumcerciana* are in shade, and isolates obtained in this study are in **bold** and blue. *Diaportheambigua* (CMW55887) was used as outgroup taxon.

Phylogenetic analyses indicated that all of the Cryphonectriaceae genera formed independent phylogenetic clades with high bootstrap values (ML > 77%, MP > 100%) both in the ML and MP analyses, with the exception of *Aurifilum*, and strains sequenced in this study formed sub-clades (Fig. 2). The partition homogeneity test (PHT), comparing the combined ITS and *BT2/BT1* loci dataset generated a value of *P* was 0.68, indicating some incongruence in the dataset of the four loci, and the accuracy of the combined data suffered relative to the individual partitions (Huelsenbeck et al. 1996; Cunningham 1997).

Further species analyses selected twenty-four *Aurifilum* isolates (Table 2). Based on the sequences of ITS, *BT2/BT1*, *TEF-1α*, *rpb2* sequences, four genotypes were generated for the 20 isolates sequenced in this study (Table 2). Sequences for two ex-type specimen strains and other of two *Aurifilum* species related to isolates obtained in this study were downloaded from GenBank (Table 1). *Celoporthecerciana* (CERC9128) was used as an outgroup taxon. The partition homogeneity test (PHT), comparing the combined ITS, *BT2/BT1*, *TEF-1α* and *rpb2* loci dataset generated a value of *P* was 1, indicating some incongruence in the dataset of the four loci, and the accuracy of the combined data suffered relative to the individual partitions (Huelsenbeck et al. 1996; Cunningham 1997). Although the *P* value was high, the sequence of four loci was combined and subjected to phylogenetic analyses. All sequences obtained for the isolates of *Aurifilum* in this study were deposited in GeneBank (Table 2). The number of taxa and characters in each of the datasets, and the summary of the most important parameters applied in the MP and ML analyses, are presented in Table 3. The six datasets were deposited in TreeBASE (http://purl.org/phylo/treebase/phylows/study/TB2:S30284?x-access-code=cf2a0ef843604b8fa4301eced72cec7f&format=html,30284).

**Table 3.** Datasets used and statistics resulting from phylogenetic analyses.

**Dataset**	**No. of taxa**	**No. of bp ^a^**	**Maximum parsimony**								
			**PIC ^b^**	**No. of trees**	**Tree length**	**CI ^c^**	**RI ^d^**	**RC ^e^**	**HI ^f^**		
ITS+BT	116	1465	4	1	6	1.000	1.000	1.000	0		
ITS	25	558	3	1	3	1.000	1.000	1.000	0		
BT	25	907	12	1	12	1.000	1.000	1.000	0		
TEF	23	266	1	1	1	1.000	1.000	1.000	0		
rpb2	23	1058	6	1	6	1.000	1.000	1.000	0		
ITS+BT+TEF+rpb2	25	2789	22	1	22	1.000	1.000	1.000	0		
**Dataset**	**Maximum likelihood**										
	**Subst. model ^g^**	**NST ^h^**	**Rate matrix**					**Ti/Tv ratio ^i^**	**p-inv**	**Gamma**	**Rates**
ITS+BT	TPM2uf+I+G	6	1.428	4.552	1.428	1.000	4.526	4.525	0.445	1.107	gamma
ITS	TrNef	6	1.000	1.389	1.000	1.000	3.247	–	0	–	equal
BT	TrN	6	1.000	2.380	1.000	1.000	5.893	–	0	–	equal
TEF	TrN	6	1.000	1.989	1.000	1.000	4.887	–	0	–	equal
rpb2	TrN+G	6	1.000	4.377	1.000	1.000	233.189	–	0	0.055	gamma
ITS+BT+TEF+rpb2	TrN	6	1.000	2.257	1.000	1.000	7.842	–	0	–	equal

^a^bp = base pairs. ^b^PIC = number of parsimony informative characters. ^c^CI = consistency index. ^d^RI = retention index. ^e^HI = homoplasy index. ^f^RC = rescaled consistency index. ^g^model = best-fit substitution model. ^h^NST = number of substitution rate categories. ^I^Ti/Tv ratio = transition/transversion ratio.

**Figure 3.**
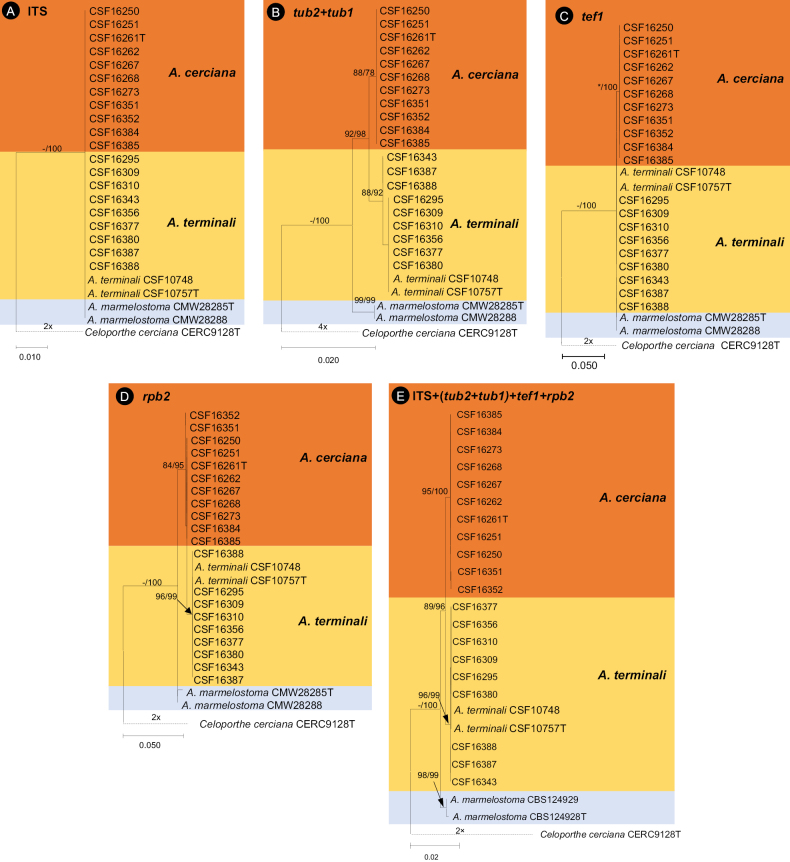
Phylogenetic trees based on maximum likelihood (ML) analyses for species in *Aurifilum***A**ITS region **B** two regions of β-tublin (BT2/BT1) **C***TEF-1α* gene region **D***rpb2* gene region **E** combination of ITS, BT2/BT1, *TEF-1α* and *rpb2* regions. Bootstrap values ≥ 70% for ML and MP (maximum parsimony) analyses are presented at branches as ML/MP. Bootstrap value lower than 70% are marked with *, and absent analysis value are marked with –. Isolates representing *A.cerciana* are in shade, and isolates obtained in this study are numbered followed CSF. *Celoporthecirciana* (CERC9128) was used as outgroup taxon.

For each of the six datasets, the MP and ML analyses generated trees with generally consistent topologies and phylogenetic relationships among taxa. Among the trees generated by the *Aurifilum* spp. single loci dataset, the *BT2/BT1*, *TEF-1α*, *rpb2* show that 20 isolates obtained in this study mainly grouped into two clades, one clade contained nine isolates cluster into a lineage with *A.terminali*, the other 11 isolates clade formed a novel monophyletic lineage that was distinct from any known *Aurifilum* sp., and was supported by high bootstrap values in these gene trees (Fig. 3B–D).

Among the *BT2*/*BT1* trees, isolate CSF16343, CSF16387, CSF16388 grouped into the lineage with *A.terminali*, and among the *rpb2* tree, isolates CSF16351, CSF16352 grouped into the novel lineage, formed a single independent branch but the bootstraps value within the clades were not significant (Fig. 3B, D), which suggests that these differences reflect intraspecific rather than interspecific variation. The combined ITS, *BT2/BT1*, *TEF-1α* and *rpb2* tree (Fig. 3E) indicated that the isolates grouped into novel lineage are putative undescribed species of *Aurifilum* (bootstrap values of the combined dataset, ML and MP: 96 and 100%).

### ﻿Morphology and taxonomy

Based on phylogenetic analyses and morphology characteristics, the isolates from Terminalia trees in southern China represent two distinct species in *Aurifilum*. Isolates CSF16295, CSF16309, CSF16310, CSF16343, CSF16356, CSF16377, CSF16380, CSF16387, CSF16388 in phylogenetic cluster with *A.terminali* (Fig. 3B–E), and isolates CSF16343, CSF16387, CSF16388 appear a branch in *BT2/BT1*, *rpb2*, and *combine* trees (Fig. 3B, D, E) in this cluster, was finally identified as *A.terminali*. The isolates in the other cluster present a novel species in *Aurifilum*, here named as *Aurifilumcerciana* sp. nov. (Fig. 3); this unknown species was described as follows:

#### ﻿ Aurifilum cerciana

Taxon classificationFungiDiaporthalesCryphonectriaceae

W. Wang & S.F. Chen sp. nov.

D4F3F5AD-9AEF-509E-B4E4-E43FA218912D

MycoBank No: 848235

Fig. 4

##### Etymology.

the name refers to China Eucalypt Research Centre (CERC), the former institution of the Research Institute of Fast-Growing Trees (RIFT), which served as the identification site for this study on Terminalia trees disease caused by *Aurifilum* spp.

##### Stromata.

**Figure 4.**
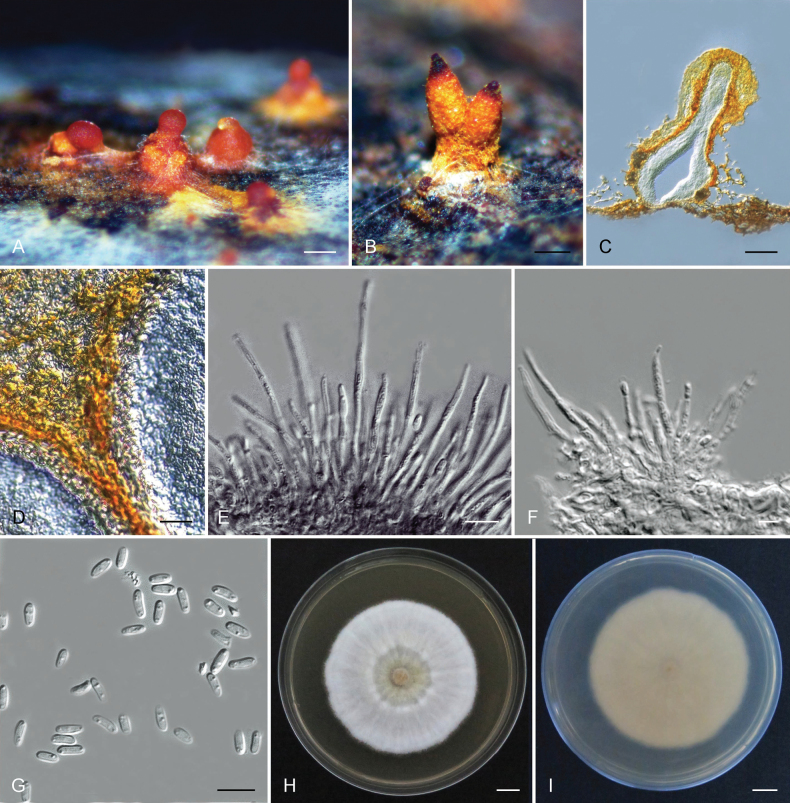
Morphological characteristics of *Aurifilumcerciana***A, B** conidiomata on the bark **C** longitudinal section through conidioma showing umber stroma **D** prosenchymatous stromatic tissue of the conidia **E** paraphyses **F** conidiophores and conidiogenous cells **G** conidia **H, I** colony of *A.cerciana* on MEA after 7 days at 25 °C **H** front **I** reverse. Scal bars: 200 µm (**A**); 100 µm (**B, C**); 10 µm (**D, E, F**); 5 µm (**G**); 1 cm (**H, I**).

No ascostromata were observed on inoculated *Eucalyptus* branch tissue, the conidiomata on the inoculated *Eucalyptus* branch tissue were superficial to slightly immersed, pulvinate, globose pyriform to various shapes without necks, blight yellow when young, orange to brown when mature (Fig. 4A, B), unilocular, 46–236 μm (av. 142 μm) diameter (Fig. 4C). Stromatic tissue prosenchymatous (Fig. 4D). Stromatic conidiomatal base was 119 – 678 μm (av. 428 μm) high above the level of the bark and 58 – 269 μm (av. 158 μm) wide. Conidiomatal necks absent. Conidiomatal locules unilocular. Conidiophores, hyaline, branched irregularly at the base or above into cylindrical cells, with or without separating septa, (11.2–)23.8–28.6(–70.2) μm (av. 26.2 μm) long, (1.7–)2.3–3.7(–6.5) μm (av. 3 μm) wide (Fig. 4F). Conidiogenous cells phialidic, cylindrical, without attenuated apices, (0.8–)1.0 – 1.8(–2.6) μm (av. 1.4 μm) wide (Fig. 4F). Paraphyses or cylindrical sterile cells, occur among conidiophores, up to 99 μm (av. 51.4 μm) long (Fig. 4E). Conidia hyaline, non-septate, oblong to cylindrical, occasionally allantoid, extend through on opening at stromatal surface as orange droplets, (3.6–)4.3–4.5(–5.7) × (1.5–)1.8(–2.2) (av. 4.4 × 1.8 μm) (Fig. 4G).

##### Culture characteristics.

Colonies on MEA are fluffy with an uneven margin, white when young, turning pale luteous to luteous after 10 days, and reverse yellow to orange-white. Optimal growth temperature 35 °C, reaching the edge of the 90 mm plates after 7 days. No growth at 5, 10 °C. After 7 days, colonies at 15, 20, 25, 30, and 35 °C reached 15.8, 45.9, 49, 50.5, and 74.4 mm, respectively.

##### Substrate.

Bark of *Terminalianeotaliala*.

##### Distribution.

Guangdong Province, China.

##### Additional materials examined.

China, Guangdong Province, Zhanjiang Region, Suixi District, Chating Town (21°16′06.97″N, 110°5′16.8432″E) from branch bark of *T.neotaliala* tree, 23 May 2019, S. F. Chen & W. Wang, holotype, CSFF2078, HMAS350333, ex-type culture CSF16261 = CGMCC3.20107; Guangdong Province, Zhanjiang Region, Suixi District, Diaolou Town (21°15′57.006″N, 110°12′26.5824″E) from twigs of *T.mantaly* tree, 25 May 2019, S. F. Chen & W. Wang, CSFF2079, HMAS350334, culture CSF16384 = CGMCC3.20108.

##### Notes.

Three species were described in the genus *Aurifilum*, including *A.marmelostoma*, *A.terminali*, *A.cerciana*. *Aurifilumcerciana* morphologically differs from *A.terminali* by the absence of conidiomatal necks (Wang et al. 2020), and differs from *A.marmelostoma* by longer paraphyses (Begoude et al. 2010). *A.cerciana* could also be distinguished from *A.terminali* and *A.marmelostoma* by growth characteristics in culture. The optimal growth temperature of *A.cerciana* is 35 °C, whereas *A.terminali* grows relatively slowly at this temperature and no growth is observed for *A.marmelostoma* (Begoude et al. 2010; Wang et al. 2020).

##### Pathogenicity tests.

**Figure 5.**
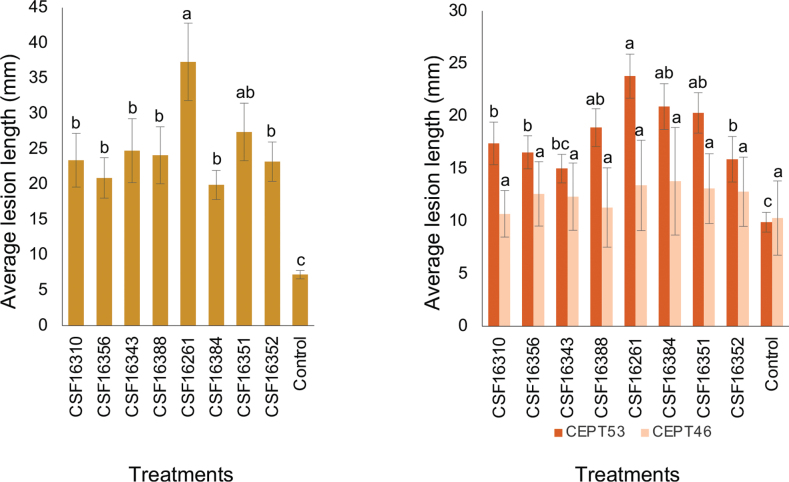
Column chart showing average lesion lengths (mm) produced by each isolate of *Aurifilum* on the branches of *T.neotaliala* (left) and two *Eucalyptus* hybrid genotypes (right). Eight isolates of *Aurifilum* were used. Vertical bars represent the standard error of the means. Different letters above the bars indicate treatments that were statistically significantly different (*P* = 0.05).

Eight isolates representing the two species of *Aurifilum* identified in this study were used to inoculate seedlings of two *Eucalyptus* hybrid genotypes, and branches of *T.neotaliala*. These include four isolates in *A.terminali* and *A.cerciana*, respectively (Table 2). Seedling stems or tree branches inoculated with *Aurifilum* isolates exhibited lesions, whereas the control group only showed wounds without any lesions. (Fig. 5). The lesions produced by *Aurifilum* species on *T.neotaliala* and *Eucalyptus* clones CEPT53 were significantly longer than the wounds on the controls (P < 0.05), whereas for the *Eucalyptus* clones CEPT46, the lesions produced by *Aurifilum* species were not significantly different (Fig. 5). The overall data revealed that *A.cerciana* and *A.terminali* have similar pathogenicity (Fig. 5). The overall data further showed that CEPT53 is more susceptible than CEPT46 to *Aurifilum* spp. (Fig. 5B). Yellow or orange fruiting structures and cankers were produced on the bark of inoculated trees within 7 weeks; these structures displayed similar characteristics of conidiomata on the Terminalia trees in the field and the re-isolated fungi from lesions share the same culture morphology with the *Aurifilum* fungi originally from the Terminalia trees in the nursery. The inoculated *Aurifilum* fungi were successfully re-isolated from the lesions but not from the control, indicating that the Koch’s postulates had been fulfilled.

## ﻿Discussion

In this study, many *Aurifilum* isolates were obtained from diseased Terminalia trees in Southern China, and two species of four genotypes belonging to *Aurifilum* were identified from two species of Terminalia. Including the new taxon, *A.cerciana* sp. nov., there are fifty-seven taxa in the Cryphonectriaceae.

In the genus *Aurifilum*, *A.marmelostoma* was the first described species, which was isolated from the bark of native *T.ivorensis* and the dead branches of non-native *T.mantaly* in Cameroon (Begoude et al. 2010), and the *A.terminali*, the second identified species, was isolated from non-native *T.neotaliala* in southern China (Wang et al. 2020). In the present study, a new species, *A.cerciana* was isolated from non-native *T.neotaliala* and *T.mantaly*, and a previously known species, *A.terminali* was isolated from *T.mantaly* too. The species *T.mantaly* was a newly reported host for *A.terminali*. Our results indicated that the *Aurifilum* species are widely distributed on non-native Terminalia trees in southern China, which is consistent with the previous hypothesis of Wang et al. (2020).

Members of the Cryphonectriaceae are well known to occur on Myrtales in Southern China. Prior to this study, six genera, including *Aurifilum*, *Celoporthe*, *Chrysoporthe*, *Chrysomorbus*, *Corticimorbus*, *Parvosmorbus* were reported infecting trees in Combretaceae, Lythraceae, Melastomataceae and Myrtaceae (All Myrtales) in southern China (Chen et al. 2010, 2011, 2016, 2018; Wang et al. 2020). Although the diversity of Cryphonectriaceae in Myrtales has been extensively studied in recent years (Chen et al. 2010, 2011, 2013a, b, 2016a, b, 2018; Wang et al. 2018, 2020; Huang et al. 2022), there is still a need for further investigation into its diversity, geographical distribution, and host range in China (Wingfield et al. 2015).

Pathogenicity test showed that all tested *Aurifilum* isolates were pathogenic to mature *T.neotaliala* and *E.grandis* hybrid genotypes of CEPT53 and CEPT46 seedlings. To clarify the threat of these pathogens to *Eucalyptus* plantations, further inoculations on mature *Eucalyptus* in the field should be conducted. Variations in pathogenicity among different individuals of the same species have been observed, with some strains showing stronger pathogenicity in different hosts. This phenomenon has also been observed in previous studies (Chen et al. 2010, 2011, 2013a, b; 2016a, b, 2018; Wang et al. 2018, 2020), and further comparison of the genetic features of these individual exhibiting differences in pathogenicity may help reveal the pathogenic mechanisms of the pathogen.

## Supplementary Material

XML Treatment for Aurifilum cerciana

## ﻿Additional information

### Author ORCIDs

ShuaiFei Chen https://orcid.org/0000-0002-3920-9982

### Data availability

All of the data that support the findings of this study are available in the main text or Supplementary Information.
